# Differential cellular recognition pattern to *M. tuberculosis* targets defined by IFN-γ and IL-17 production in blood from TB + patients from Honduras as compared to health care workers: TB and immune responses in patients from Honduras

**DOI:** 10.1186/1471-2334-13-125

**Published:** 2013-03-06

**Authors:** Nancy Alvarez-Corrales, Raija K Ahmed, Carol A Rodriguez, Kithiganahalli N Balaji, Rebeca Rivera, Ramakrishna Sompallae, Nalini K Vudattu, Sven E Hoffner, Alimuddin Zumla, Lelany Pineda-Garcia, Markus Maeurer

**Affiliations:** 1 Escuela de Microbiología, Universidad Nacional Autónoma de Honduras (UNAH), Tegucigalpa, Honduras; 2 Department of Microbiology, Tumor and Cell Biology (MTC), Karolinska Institutet, Stockholm, Sweden; 3 Swedish Institute for Communicable Disease Control (SMI), Stockholm, Sweden; 4 Department of Microbiology and Cell Biology, Indian Institute of Science, Bangalore, India; 5 Department of Pathology, University of Iowa, Iowa city, IA, USA; 6 Department of Immunobiology, Yale University, New Haven, CT, USA; 7 Department of Infection, University College London Medical School, Windeyer Institute of Medical Sciences, London, UK; 8 Center for allogeneic stem cell transplantation (CAST), Karolinska University Hospital, Stockholm, Sweden; 9 Department of Laboratory Medicine, Division of Therapeutic Immunology, Karolinska Institutet, Stockholm, Sweden

**Keywords:** T-cells, M. tuberculosis, TB, Antigen-recognition, Biomarkers

## Abstract

**Background:**

A better understanding of the quality of cellular immune responses directed against molecularly defined targets will guide the development of TB diagnostics and identification of molecularly defined, clinically relevant *M.tb* vaccine candidates.

**Methods:**

Recombinant proteins (n = 8) and peptide pools (n = 14) from *M. tuberculosis* (*M.tb*) targets were used to compare cellular immune responses defined by IFN-γ and IL-17 production using a Whole Blood Assay (WBA) in a cohort of 148 individuals, i.e. patients with TB + (n = 38), TB- individuals with other pulmonary diseases (n = 81) and individuals exposed to TB without evidence of clinical TB (health care workers, n = 29).

**Results:**

*M.tb* antigens Rv2958c (glycosyltransferase), Rv2962c (mycolyltransferase), Rv1886c (Ag85B), Rv3804c (Ag85A), and the PPE family member Rv3347c were frequently recognized, defined by IFN-γ production, in blood from healthy individuals exposed to *M.tb* (health care workers). A different recognition pattern was found for IL-17 production in blood from *M.tb* exposed individuals responding to TB10.4 (Rv0288), Ag85B (Rv1886c) and the PPE family members Rv0978c and Rv1917c.

**Conclusions:**

The pattern of immune target recognition is different in regard to IFN-γ and IL-17 production to defined molecular *M.tb* targets in PBMCs from individuals frequently exposed to *M.tb.* The data represent the first mapping of cellular immune responses against *M.tb* targets in TB patients from Honduras.

## Background

9.4 million individuals are newly diagnosed with TB and two billion people are latently infected with *M.tb* worldwide; twenty two ‘High Burden Countries’ (HBC) account for 80% of TB cases. Brazil, the only HBC in America, accounts for 35% of TB cases within the region [1-3]. Honduras ranks number eight on the list of countries with a high TB burden in Latin America and second in Central America [3,4]. 2901 TB cases were identified in Honduras during 2010, with an estimated incidence rate of 51/100,000 individuals [5]. The majority of patients with TB reside in three regions, i.e. Region Metropolitana de Cortes, the Region Departamental de San Pedro Sula and the Region Metropolitana de Tegucigalpa. Several factors, i.e. poor nutrition, HIV-*M.tb* co-infection, chronic (non-infectious) diseases, overcrowding, drug and alcohol abuse, affect the quality and magnitude of immune responses and subsequently the clinical course of TB [6].

Bacteriological diagnosis for pulmonary (and extra-pulmonary) TB in Honduras relies on smear microscopy-acid fast staining (AF-S), sputum culture on Löwenstein Jensen solid media and drug susceptibility testing (DST). TB diagnosis is supported by clinical findings (e.g. weight loss, coughing), individual patient history, epidemiology and X-rays. The tuberculin skin test (TST) is less frequently performed. The interferon gamma release assay (IGRA) is used for case finding in non-endemic countries as well as a corroborative test in specific populations such as children, patients with extra-pulmonary TB or immune-compromised individuals [7-9], IGRAs are not used to differentiate between active and latent TB. Therefore, there is still an unmet need for novel diagnostic tests to reliably diagnose extra-pulmonary TB, to differentiate between latent *vs* active TB or to indicate ‘immune protection’ and effective immune-surveillance in patients with latent TB. The testing of IFN-γ as well as IL-17 in anti-*M.tb* immune responses is biologically and clinically relevant. Both cytokines are involved in the recruitment of neutrophils, granuloma formation and in anti-*M.tb* directed immune responses [10]; diminished Th1 and Th17 responses appear to be associated with higher rates of extrapulmonary TB [11]; vice versa, expression of SOCS3 is associated with increased IL-17 production along with T-cell exhaustion (in peripheral blood cells from patients with TB [12].

Not only the nature of the immune responses, defined by cytokine production, yet also the nature of the *M.tb* encoded targets may determine the strength and magnitude of the anti-*M.tb* response. Cellular immune recognition of *M.tb* antigens, defined by cytokine production, may reflect preferential expression of *M.tb* proteins during the active and dormant phase of the infection [13-16]. The aim of this study was to compare *M.tb* specific cellular immune responses in blood from individuals with active pulmonary (symptomatic) TB and individuals who have been frequently exposed to *M.tb* in response to antigens preferentially expressed by active and dormant *M.tb.*

## Methods

### Study site and subjects

The Honduran population receives (after birth) BCG vaccination since 1977. Different BCG vaccine strains have been used, the current BCG is provided by the Serum Institute, India, through WHO/UNICEF/GAVI. Honduras has a considerable TB incidence and limited resources in health care structures, including X-ray facilities. The definition of a “clean” and well defined (non-TB+) control group has therefore been challenging. The TST was not implemented in this study, since it is not routinely performed due to high variability in cut off interpretations and subsequent variant clinical decision making. 148 subjects were enrolled in the study between August 2008 and May 2010 in Tegucigalpa, Honduras at Instituto Nacional Cardiopulmonar (INCP) and at the Health Center El Manchen. The median age of the participants was 49 years, 62% of the study participants were female. Each subject was recruited after informed consent; HIV-testing and counseling was offered to each participant. A rapid HIV-1/HIV-2 antibody test (Abbott Laboratories) was used to screen for HIV infection. Clinical data were collected from hospital records and through structured interviews. Heparinized blood and sputum samples were obtained from each donor after written informed consent, morning (instant) sputum samples were collected after the interview and inclusion in the study. For hospitalized patients, sampling was performed in the morning as part of the diagnostic workup (3 days morning samples). The population was divided into three groups based on their clinical status and bacteriological results: Group1: TB + (n = 38) (*M.tb* culture and AFS positive, pulmonary TB) prior to initiation of DOTS; Group 2: TB- (n = 81) respiratory symptomatic patients (asthma, non-TB pneumonia, chronic-obstructive pulmonary disease, lung cancer, pharyngitis). Both outpatients and inpatients (in order to rule out TB, *M.tb* culture and AFS negative) were included in the Group 2 patients. Group 3: TB- (n = 29) health care workers from the TB units, exposed to *M.tb* (*M.tb* culture and AFS negative, no clinical signs of TB or any respiratory symptoms). LTBI was not discriminated between groups 2 and 3; however, the IGRA test was performed in both groups. All subjects tested HIV-negative. The study protocol was approved by the Institutional and National Ethical Committee, Instituto Nacional Cardiopulmonar and Comite de Etica en Investigación Biomédica (No. IRB 00003070).

**Antigens** used for T-cell stimulation assays are listed in Table 1. Pools of 15-mer long peptides, overlapping by 7 amino acid residues (covering the entire protein), were synthesized by JPT Peptide Technologies, Berlin, Germany. Synthetic peptides and recombinant protein (purity > 85%) were used at final concentration of 1 μg/ml and 5 μg/ml respectively. The antigens Rv3804c, Rv1886c, Rv0288 and Rv0959 were kindly provided by the AERAS Global TB Foundation (AERAS, Rockville, USA). Recombinant proteins Rv3875 and Rv3874 were purchased from Statens Serum Institute (SSI, Copenhagen, Denmark). The recombinant PPE-proteins Rv0754, Rv0978c and Rv1917c were produced by Professor K. N. Balaji, Bangalore, India [55,62-64]. A mixture of Staphylococcal Enterotoxin A and B, (SEA/SEB; 10 ng/mlSigma Aldrich, USA) was used as the positive control for T-cell reactivity.

**Table 1:** **Summary of *M.tb *test targets**

**Peptides**				
**Gene locus**	**RefSeq**	***M.tb *Antigens**	**aa**	**Comment**
Rv0447c	NP854118 (Pool 1)	Probable cyclopropane fatty acyl phospholipid synthase.	427	Methyl transferase activity. Cyclopropane fatty acyl phospholipid synthase activity. Lipid biosynthetic process [17,18]
Rv2940c	YP_976584 (Pool 2)	Mycocerosic acid synthase	2111	Lipid biosynthetic process. Oxido-reduction and transferase activity, Cofactor binding. Location at the cell wall. [17,19-23]
Rv3347c	YP_177963 (Pool 3)	PPE family protein	3157	Function unknown. Gly-Ala-Asn rich protein, interacts with the host system by inhibition of antigen processing. [18,23-26]
Rv2453c	CAA16030 (Pool 4)	Probable molybdopterin-guanine dinucleotide biosynthesis Protein A	201	Molybdenum cofactor biosynthesis. Molecular function as GTP binding. Located at the cytoplasm membrane. [18,23,24,27]
Rv1886c	CAB10044 (Pool 5)	Antigen 85B	325	Fibronectin binding protein. Acyltransferase activity. Secreted protein, also located at cell wall, plasma membrane. [18,28-31]
Rv1690	CAB10947 (Pool 6)	Probable lipoprotein	127	Putative uncharacterized protein. Protein binding, cellular component plasma membrane.[18,24,32]
Rv3019c	CAA16104 (Pool 7)	ESAT-6 like protein	96	Belongs to ESAT-6 (esx) family, Protein-protein interaction [18,24]
Rv2957	CAB05419 (Pool 8)	PGL/p-HBAD biosynthesis glycosyltrans-ferase MT3031	256	Glycosyl transferase activity, transferring hexosyl groups. Glycolipid biosynthetic function. Identified as a drug target. [18,24,30,33,34]
Rv1085c	CAA17201 (Pool 9)	UPF0073 membrane protein	242	Belongs to the UPF0073 (HIy-III) family. Cytolysis. Sub cellular location in cell membrane [18,24]
Rv0066c	CAA16247 (Pool 10)	Isocitrate dehydrogenase, NADP-dependent- icd2.	745	Oxidoreductase function. NAD or NADH binding. Isocitrate dehidrogenase (NADP+) activity. Magnesium ion binding. Protein homodimerization. [18,22-24,35,36]
Rv2958c	CAB05418 (Pool 11)	PGL/p-HBAD biosynthesis glycosyltrans-ferase	428	Glycolipid biosynthetic process, pathogenesis, Glycosyl transferase activity. Immune evasion or - tolerance [18,22,24,33,37]
Rv2962c	CAB05415 (Pool 12)	PGL/p-HBAD biosynthesis rhamnosyl-transferase	449	Glycolipid biosynthetic process, pathogenesis. Glycosyl transferase activity. Evasion or tolerance concerning to the host immune response. [18,22,24,30,33,37]
Rv3804c/1886	CAA17868/CAB10044	Ag85A/Ag85B Fibronecting binding protein peptide pool	338/325	Belongs to the Ag85 family, contains Esterase D. Possesses mycolyl transferase activity. [28,29,38-41]
**Proteins**				
Rv3804c	CAA17868	Ag85A. Secreted antigen 85A. Mycolyl transferase 85A. fbpA. Ag85 complex	338	Responsible for high affinity of mycobacteria to fibronectin. Possesses mycolyl transferase for biogenesis of trehalose dimycolate. [22,23,29,30,38-40,42-44]
Rv1886c	CAB10044	Ag85B. Secreted antigen 85B. Mycolyl transferase 85B. fbpB. Ag85 complex	325	Fibronectin binding protein. Acyltransferase activity. Response to antibiotic. Secreted protein also located at cell wall, extracellular region. [28-30,36,38,40,41,45]
Rv3875	CAA56099	ESAT-6. 6 KDa early secretory antigenic target. esxA	95	Function unknown. Elicit high level of IFNgamma during the first phase of protective immune response. Secreted protein, cell wall and cytoplasm. [46-54]
Rv3874	CAA17966	CFP-10. 10 KDa culture filtrate antigen	100	ESAT-6 like protein esxB. Forms a tight 1:1 complex with EsxA. Protein binding. Host cell surface binding. Pathogenesis. Protein secretion. [22,47-49,51,53,54]
Rv0754	CAE55319	PE_PGR11. PE-PGRS family protein	584	Member of *M.tb* PE family. PGRS subfamily gly-rich proteins. Unknown function. Protein existence predicted. [18,21]
Rv0978c	CAE55343	PE_PGR17. PE-PGRS family protein	331	Member of *M.tb* PE family. PGRS subfamily gly-rich proteins. Unknown function. Protein existence predicted. [18,55,56]
Rv1917c	CAE55440	PPE34. PPE family protein	1459	Member of *M.tb* PPE family. Glycin rich proteins. Unknown function. [18,57]
Rv0288	CAA17363	TB10.4. Low molecular weight protein. (M.tb)	96	Belongs to theESAT-6 (esx) family. Molecular function protein binding. Involved in virulence. Immunogenic. [18,24,58-61]

### Whole blood assay

The whole blood assay (WBA) was used to determine IFN-γ and IL-17 production in response to *M.tb* antigens. Venous whole blood was obtained using heparinized blood collection tubes and diluted 1:2.5 in RPMI 1640-medium supplemented with 1% Hepes, 0.5% Penicillin/ (100 IU/ml) and streptomycin (10 mg/ml), (Gibco Invitrogen). 100 μl of diluted blood was added into 96-well round bottom plates (Nunc, Roskilde, Denmark) in duplicate wells pre-coated with the specific antigen diluted in 100 μl medium. Cultures were incubated at 37°C, 5% CO_2_. After 7 days, 75 μl of cell culture supernatant was removed from each duplicate well, pooled, and stored at −80°C until IFN-γ and IL-17 were determined by ELISA.

### IFN-γ and IL-17A determination

IFN-γ and IL-17A (IL-17) in cell culture supernatants was measured by ELISA (Eli-pair DIACLONE, Biosite, Stockholm, Sweden). The assays were performed according to the manufacturer’s instruction. In brief, Nunc-Immuno™ Maxisorp 96-well plates (Nunc, GTF, Stockholm, Sweden) were coated with the specific capture antibody overnight at 4°C. The plates were then washed with PBS containing 0.05% tween-20 and then blocked with PBS containing 5% bovine serum albumin (BSA, Karolinska Hospital, Stockholm, Sweden). Supernatants collected from WBA (150 μl) were thawed and diluted (1:1.47 in IFN-γ [75 μl] and 1:1.91 for IL-17 [55 μl] with PBS containing 1% BSA. Values were multiplied by their corresponding dilution factor, background from un-stimulated medium control were subtracted from each antigen response both for IFN-γ and IL-17; the cytokine concentration was expressed in pg/ml. The detection range for IFN-γ was 400–7 pg/ml and for IL-17 100–3.1 pg/ml. An additional standard IFN-γ recombination protein (purchased from R&D, Minneapolis, MN, USA) was used as an internal control to gauge for differences between ELISA assay performances.

### Interferon gamma release assay (IGRA)

Quantiferon TB-Gold in tube (QFT-GIT) (Cellestis, Copenhagen, Denmark) [65], was performed. Briefly, 1 ml of venous blood was collected directly into three tubes containing TB-specific antigens, mitogen and nil control. Tubes were incubated at 37°C, 5% CO_2_ for 16 to 24 hours before centrifugation at 3000 g for 15 min. The plasma was collected and stored at +4°C until IFN-γ detection within two weeks. ELISA was performed as described by the manufacturer, in brief, 50 μl of conjugate was added as well as 50 μl of test sample; diluted standards were added to designated wells followed by 2 hours incubation. After washing, 100 μl of substrate solution were added to wells and incubated for 30 minutes, followed by 50 μl of enzyme stopping solution. OD values were calculated using the Software A-QTF-2.5-02 2.5, a free online source provided by Cellestis validating the assay by internal quality controls and setting the cut off for positive results at >0.35 International units (UI)/ml.

### Sputum based acid-fast staining (AF-S) and Löwenstein Jensen (LJ) culture

Acid fast staining (Ziehl-Neelsen technique) was performed to visualize the acid-fast bacilli in sputum samples. Samples were further cultured in duplicate at 37°C and periodically revised up to 8 weeks (Löwenstein Jensen selective media produced at the Research lab). In 14/148 individuals, no cultures could be initiated due to inadequate sputum procurement; the contamination rate of cultures was 2/132. Positive cultures were confirmed using standard biochemical tests, i.e. niacin, reduction of nitrates and catalase activity [66]. Quality control of Löwenstein-Jensen media was routinely performed using *M.tb* control strains at the microbiology department of the Hospital.

### Statistical analysis

Data were explored using dotplot and barplot analysis. A t-test was used to examine differences in IFN-γ and IL-17 production between groups. An adjustment for multiplicity was applied on the resulting p-values from the tests, only those antigens that showed significant difference at 5% level were discussed. We used the R software pairwise t-test function to compare the treatment group means with pooled Standard Deviation. The ANOVA test was performed in order to confirm significant differences between groups as well as a pairwise t-test and the Holm adjustment method for multiple corrections.

## Results

### Increased IFN-γ production to *M.tb* antigens in blood from health care workers exposed to *M.tuberculosis* as compared to TB patients

**Figure 1:**
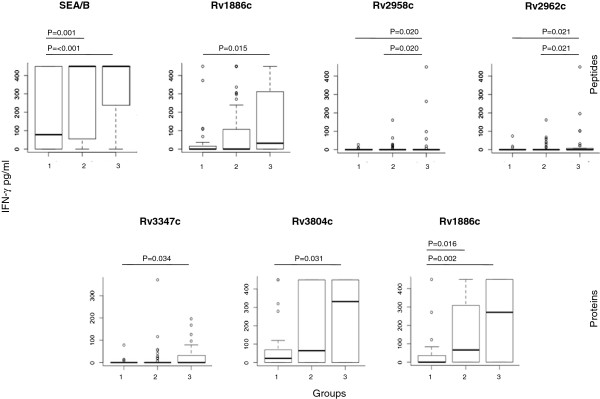
**IFN-γ boxplots of immune reactivity in blood stimulated with antigens/peptides that showed significant differences between groups (group 1, TB + patients, group 2, TB- other pulmonary diseases, group 3, frequent *M.tb *exposure, yet clinically healthy).** Antigens were identified after testing a broad panel of *M.tb* antigens (see Table 1). Only statistically significant different IFN-γ responses are shown. Strong recognition of Ag85B (Rv1886c), PGL/p-HBAD biosynthesis glycosyltransferase (Rv2958c), PGL/p-HBAD biosynthesis rhamnosyl-transferase (Rv2962c) and PPE55 (Rv3347c). Recombinant proteins Ag85A (Rv3804) and Ag85B (Rv1886c) were differentially recognized among TB + and TB- groups defined by IFN-γ release. The thick lines (inside the boxplots) represent median values.

IFN-γ production in response to *M.tb* target antigens (overview see Table 1) was analyzed in blood from the study participants (n = 148). We identified a significant difference in IFN-γ production between the groups (see Figure 1, Table 2 and Additional file 1: Table S1a-c) i.e. a different magnitudes of IFN-γ production in response to (peptide cocktails) Rv2958c, Rv2962c, Rv3347c, Rv3804c, and Rv1886c (protein) between groups 1 (TB+) and 3 (health care workers); the antigens Rv2958c and Rv2962c were differentially recognized between group 2 (non-TB pulmonary diseases) and 3 (health care workers).

**Table 2:** Statistically different cytokine production in groups 1(TB patients), 2 (patients with other pulmonary diseases) and 3 (negative TB cases but highly exposed)

**Antigen**	**Groups**	**P-value**
SEA/B	Group1 vs Group2	0.001
SEA/B	Group1 vs Group3	< 0.001
Rv1886c	Group1 vs Group3	0.015
Rv2958c	Group1 vs Group3	0.020
Rv2958c	Group2 vs Group3	0.020
Rv2962c	Group1 vs Group3	0.021
Rv2962c	Group2 vs Group3	0.021
Rv3347c	Group1 vs Group3	0.034
Rv3804c	Group1 vs Group3	0.031
Rv1886c	Group1 vs Group2	0.016
Rv1886c	Group1 vs Group3	0.002

Immune cells from individuals in group 2 (TB-, other respiratory diseases) and group 3 (exposed to TB, no clinical TB uninfected) produced significantly higher levels of IFN-γ in response to stimulation with Rv1886c (Ag85B) Rv2958c (glycosyl-transferase) and Rv2962c (pHBAD biosynthesis rhamnosyltransferase). The recombinant protein antigens Rv3804c (Ag85A) and Rv3347c (PPE family member) induced the strongest IFN-γ production in blood from group 3 (*M.tb* exposed individuals) as compared to TB + individuals (group 1). A head-to-head comparison between overlapping peptides for Rv1886c (Ag85B) and the recombinant protein (Table 2) yielded a similar trend (p = 0.015) for differences between group 1 and 3 (using peptides as the assay target) as well as for testing the recombinant protein (comparison between groups 1 and 3, p = 0.002).

### A different *M.tb* target recognition pattern defined by IL-17 production

**Figure 2:**
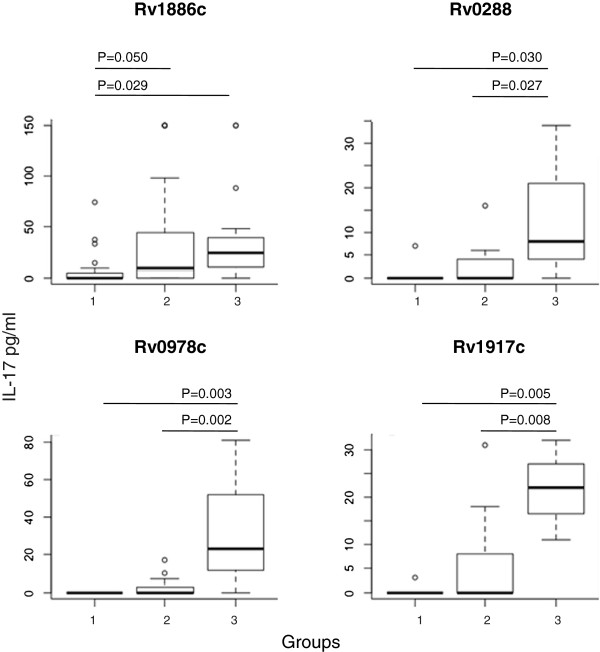
**IL-17 production shows a different pattern of cellular immune recognition as compared to IFN-γ. IL-17 production in blood was statistically different in response to the antigens Ag85B (Rv1886c), TB10.4 (Rv0288), PPE - PGR11 (Rv0978c) and PPE34 (Rv1917c).** The thick lines (inside the boxplots) represent median values. Identical patient cohorts as in Figure 1.

A more recent study revealed differences between IFN-γ and IL-17 production in response to *M.tb* antigens [67]. We selected therefore 14 *M.tb* antigens for further analysis and tested IL-17 production in response to the antigens Rv0447, Rv1886c, Rv3019c, Rv2957, Rv2958c, Rv2962c (peptides) and the recombinant proteins Rv3804c, Rv1886c, Rv3874, Rv3875, Rv0288, Rv0754, Rv0978a and Rv1917 (see Figure 2, Table 3, Additional file 1: Table S1a-c). We were able to demonstrate significantly different IL-17 production in response to Rv1886c (Ag85B) in individuals from group 2 (non TB, other respiratory disease) and 3 (health care workers, exposed to TB) as compared to individuals in group 1 (pulmonary TB). Immune cells from individuals in group 2 and group 3 produced more IL-17 in response to Rv0978c (PPE family member), Rv0288 (TB10.4) and Rv1917c (PPE family member) as compared to blood from individuals with TB (group 1, patients with pulmonary TB). The highest IL-17 production was identified in blood from exposed individuals directed against the antigens listed above.

**Table 3:** **Distribution of IFN-γ levels for the antigens associated with statistically different cytokine production between groups at 5**% **level**

**Antigen**	**Groups**	**P-value**
Rv1886c	Group1 vs Group2	0.050
Rv1886c	Group1 vs Group3	0.029
Rv0288	Group1 vs Group3	0.030
Rv0288	Group2 vs Group3	0.027
Rv0978c	Group1 vs Group3	0.003
Rv0978c	Group2 vs Group3	0.002
Rv1917c	Group1 vs Group3	0.005
Rv1917c	Group2 vs Group3	0.008

Differences in IFN-γ between groups were examined using a t-test; adjustment for multiplicity was applied on the resulting p-values from the tests. (group 1: TB+, AFS and culture positive, 2: other pulmonary diseases, no exposure records to TB, 3: health care workers, frequently exposed to *M.tb*, yet clinically healthy). Only statistically relevant differences are listed.

### Analysis between QFT-GIT and bacteriology

We analyzed the cytokine responses in blood from individuals stratified by the QFT-GIT (see online Additional file 1: Table S2-4 and Figures S1 and S2) based on the acid fast stain result, culture positivity and previous history of TB). Immune cells from individuals with AFS + *M.tb* + culture showed decreased IFN-γ responses to the positive (SEA/SEB) controls. ESAT-6 and CFP-10 immune reactivity was significantly higher in blood from individuals with AFS- and negative culture results– yet with a positive QFT-GIT for IFN-γ production (and for IL-17 production in response to Rv3874, CFP10). Significant differences concerning cytokine response patterns could be identified in blood from patients with TB (AFS+, QFT-GIT + and culture+) versus blood from health care workers in response to Rv3804c (Ag85A, IFN-γ p = 0.009 as well as for IL-17, p = 0.019) and Rv2962 (pHBAD, rhamnosyl-transferase, p = 0.042 for IL-17 production) (Additional file 1: Tables S2-4, Additional file 1: Figures S1 and S2).

## Discussion

Tuberculosis is the result of a dynamic host-pathogen relationship. Anti-*M.tb* directed immune responses may be associated with immune-protection, yet also with immune-pathology, as shown for IL-17-driven cellular immune responses. IL-17 – associated immune responses can be protective or harmful in TB and may lead to lung tissue damage along with massive inflammation and influx of neutrophils [68]. Most immune assays aim to gauge *M.tb* responses measure IFN-γ, which is indispensable to contain *M.tb,* yet other immune effector cytokines, e.g. IL-17, may also be instrumental in immune protection. Further evidence for the beneficial role of IL-17 is provided from a pre-clinical model: a recombinant BCG vaccine expressing listeriolysin, leads to a statistically significant different IL-17 (yet not IFN-γ) production [69] in a murine *M.tb* challenge model.

We were able to show that IFN-γ mediated responses showed strong cellular recognition of Rv1886c (Ag85B), Rv3804c (Ag85A), Rv2958c, Rv2962c (enzymes associated with lipid-alteration) and Rv3347 (PPE family member) in blood from individuals who have been exposed to *M.tb*, yet are clinically healthy. The data are compiled for review in Table 4 concerning the detailed clinical diagnoses of individuals enrolled in the study; Table 5 summarizes the molecularly defined test antigens leading to IFN-γ and IL-17 production. Decreased IFN-γ production in blood from individuals with TB (AFS and culture positive) may in part be related to ‘anergy’, which is reflected in significantly lower IFN-γ responses to the positive control stimulus (SEA/SEB, Figure 1). This notion is further supported by a more detailed examination of patient subpopulations (see online Additional file 1: Figure S1 and Table S2). Only blood from individuals with a AFS+, culture+, QFT-GIT + profile showed decreased IFN-γ production, this was not found to be true for individuals with presumably latent TB (based on exposure history, QFT-GIT+, yet culture - AFS-).

**Table 4:** **Description of the general population, clinical characteristics, microbiology and immunological test result**s

**Group**	**QTF-GIT**	**AF-S**	**Culture**	**X-ray**	**Gender**	**Comments**
**TB + HIV- n = 38**	Pos: 23	Pos: 26	MTB: 23	altered: 23	Female: 17	Pulmonary TB (26), Pleural TB (3) extra-pulmonary TB (9)
	Neg: 10	Neg:12	Neg: 11	non altered: 5	Male: 21	
	Ind: 5	No data: 0	Cont: 1 No data: 3	no data: 10		
**TB-HIV- n = 81**	Pos: 24	Pos: 0	Neg: 79	altered: 33	Female: 56	Allergy (1) asthma (17), EPOC (20), (non-TB) pneumonia (8), lung cancer (4), (non-TB) pleural effusion (1), bronchitis (5), other (25) otitis, rhinitis, influenza, diabetes, heart disease.
	Neg: 54	Neg: 81	Cont: 1	non altered: 7	Male: 25	
	Ind: 3	No data: 0	No data: 1	no data: 41		
**TB-HIV- n = 29**	Pos: 11	Pos: 0	Neg: 19	altered: 10	Female: 19	Nurses and TB/HIV health care workers (19), previous TB with successful treatment regimen in the past (at least 2 years) but no current TB (10)
	Neg: 17	Neg: 19	No data: 10	non altered: 2	Male: 10	
	Ind: 1	No data: 10		no data: 17		

Ind: indeterminate; Neg: negative, Pos: positive.

**Table 5:** Compilation of the antigen-specific response analysis

**Antigen**	**Group 1**		**Group 2**		**Group 3**	
	IFN-γ (%)	IL-17 (%)	IFN-γ (%)	IL-17 (%)	IFN-γ (%)	IL-17 (%)
**Rv0447c**	10/38 (26)	10/38 (26)	24/81 (30)	17/81 (21)	12/29 (41)	11/29 (38)
**Rv2940c**	3/38 (8)		8/81 (10)		3/29 (10)	
**Rv3347c**	5/38 (13)		12/81 (15)		12/29 (41)	
**Rv2453c**	4/38 (11)		11/81 (14)		9/29 (31)	
**Rv1886c**	14/38 (37)	10/38 (26)	37/81 (46)	27/81 (33)	15/29 (52)	14/29 (48)
**Rv1690**	6/38 (16)		18/81 (22)		8/29 (28)	
**Rv3019c**	8/38 (21)	10/38 (26)	29/81 (36)	37/81 (46)	9/29 (31)	16/29 (55)
**Rv2957**	7/38 (18)	11/38 (29)	30/81 (37)	33/81 (41)	11/29 (38)	12/29 (41)
**Rv1085c**	6/38 (16)		11/81 (14)		4/29 (14)	
**Rv0066c**	3/38 (8)		14/81 (17)		4/29 (14)	
**Rv2958c**	4/38 (11)	14/38 (37)	10/81 (12)	31/81 (38)	7/29 (24)	13/29 (45)
**Rv2962c**	3/38 (8)	10/38 (26)	10/81 (12)	32/81 (40)	8/29 (28)	15/29 (28)
**Rv1886c**	12/25(48)	12/25 (48)	40/65 (62)	47/65 (72)	11/17 (65)	15/17 (88)
**Rv3804c**	15/25 (60)	15/25 (60)	40/65 (62)	44/65 (68)	12/17 (71)	12/17 (71)
**Rv3874**	13/20 (65)	8/20 (40)	32/48 (67)	25/48 (52)	13/14 (93)	7/14 (50)
**Rv3875**	9/20 (45)	8/20 (40)	24/48 (50)	11/48 (23)	6/14 (43)	5/14 (36)
**Rv0288 pep**	3/11 (27)		3/21 (14)		0/3 (0)	
**Rv3804c/Rv1886c**	3/11 (27)		3/21 (14)		0/3 (0)	
**Rv3875/3874**	7/9 (78)		6/21 (29)		0/5 (0)	
**Rv0754**	3/5 (60)	1/5 (20)	1/17 (6)	5/17 (29)	1/3 (33)	3/3 (100)
**Rv0978**	2/5 (40)	0/5 (0)	4/17 (24)	5/17 (29)	0/3 (0)	2/3 (66)
**Rv1917**	2/5 (40)	1/5 (20)	2/17 (12)	7/17 (41)	0/3 (0)	3/3 (100)
**Rv0288**	1/5 (20)	1/5 (20)	1/17 (6)	6/17 (35)	0/3 (0)	2/3 (66)

Group 1, TB + patients, group 2, TB- other pulmonary diseases, group 3, frequent *M.tb* exposure, yet clinically healthy.

Of interest is the quite different cellular reactivity concerning *M.tb* target pattern recognitions if IFN-γ and IL-17 are analyzed. Ag85B (Rv1886c) showed a similar trend concerning T-cell recognition, both for IFN-γ and IL-17 production (see Figures 1 and 2). Yet the antigen Rv0288 (TB10.4) and two PPE family members (Rv0978c, Rv1917c) exhibited only statistical differences in IL-17 responses, yet not concerning the capacity to induce IFN-γ production. *Vice versa*, the PPE family member Rv3347c, the enzymes Rv2958c (glycosyl-transferrase) and Rv2962 (rhamosyl-transferase) showed only differences in IFN-γ production between the populations (groups 1, TB + and 3, health care workers). The data consolidate our previous observations in a population from Belarus, where cellular immune responses, defined by IFN-γ production, were more frequently directed against the antigen glycsoltransferase (Rv2958c) in healthy individuals frequently exposed to *M.tb*[70]. The gene product *Rv2958c* adds a second rhamnosyl unit and a fucosyl residue to form the species-specific triglycosyl appendage of PGL-tb and *p-*HBAD. A differential expression of *Rv2958c* in BCG vaccine strains has been speculated to be associated with different levels of protection from TB [71]. The data reported in the current study consolidates also the strong immune recognition of Ag85A in blood from individuals after BCG vaccination or *M.tb* exposure [72].

Several explanations may apply for the differential recognition patterns defined by IFN-γ and IL-17 production: Rv2958c and Rv2962 were tested as overlapping peptides; some peptides may be degraded and therefore not efficiently processed and presented to T-cells; the antigen processing and presentation of peptides may be different as compared to recombinant proteins. We produced in the meantime an (LPS-free) Rv2958c protein which leads as well as to IL-17 production in blood from TB + individuals (our unpublished data), suggesting that IL-17 production may require the intact protein structure and subsequent cellular processing and presentation.

Other cellular mechanism may be important to initiate IL-17 production, i.e. the priming and activation of antigen presenting cells which will subsequently present the recombinant target protein to antigen-specific T-cells. For instance, the PPE family members (tested in the current panel) have been shown to mature dendritic cells via TLR-2 stimulation (in a murine system) leading to a different quality of antigen-presentation and expansion of antigen-specific immune cells [62]. A similar observation has been reported for differences in BCG and rBCG, expressing listeriolysin; ‘components’ released from rBCG in the cytosol of macrophages, may lead to a different array of pathogen-associated signaling patterns leading to stimulation of antigen-presenting cells and subsequent expansion of Th17+ immune cells [69]. It could very well be that the PPE family members described in the current report lead to different IFN-γ/IL-17 production via activation of antigen-presenting cells (see Figure 2).

Of interest is the strong recognition, defined by IL-17 production, of TB10.4 (Rv0288c) and Ag85B (Rv1886c), two components of several TB vaccine candidates, in blood from healthy TB- exposed individuals. A similar, statistically significant trend, was found to be true for IL-17 production in response to the PPE family members Rv0978c (p = 0.003, difference between groups 1 and 3) and Rv1917c (p = 0.008, difference between groups 1 and 3). Both proteins signal via TLR2 and are able to mature dendritic cells [62]. Future studies will show whether recombinant target proteins, used in the current study, are able to induce critical feedback regulators that would preferentially expand IL-17 –producing immune cells [12]. The strong TB10.4 recognition in blood from healthy individuals appears to be in contrast to the study of Sutherland and coworkers who reported a significant difference in IFN-γ production, yet not IL-17 production, by comparing cytokine responses in blood from TB + cases (West Africa) and a TST- control cohort [67]. Several reasons may account for these differences, i.e. a different exposure history of the test population to *M.tb* and/or environmental mycobacteria and subsequent expansion of IL-17+ producing immune cells. Not only exposures to mycobacterial species, yet other (environmental pathogens) may contribute of shaping the immune response leading to preferential IL-17 production, i.e. natural killer, natural killer T-cells, lymphoid tissue inducer and TCRγδ + T-cells are contributing to IL-17 production (for review see [73]). Future studies may therefore need to dissect the role of the cell source of IL-17 production in response to *M.tb* targets and require the stratification of immune response analysis based on the distribution of immune cells subsets (T-cells, NK, NKT, TCRγδ + T-cells) in the test samples.

## Conclusions

In summary, we report for the first time the cellular immune recognition pattern against *M.tb* in a clinically defined Honduran population characterized by differential immune recognition patterns in regard to IFN-γ and IL-17 production. The limitations of the current study are the number of study participants, the difficulty to obtain age and sex-matched control individuals, to challenge to gauge and control for multiple *M.tb* exposures and MOTT, as well as the challenge of defining latent TB infection in general, particularly in a resource-restrained country. Of particular interest is the antigen Rv2958, which is currently being evaluated as part of a new TB vaccine from our group in pre-clinical models. Screening of *M.tb* exposed, yet clinically healthy, individuals (as compared to TB + patients and non-*Mtb* exposed control cohorts, such as those described in the current study) may help to better identify immunological markers which help to define *M.tb* exposure and immune protection.

## Competing interest

The authors declare that they have no competing interests.

## Author’s contributions

NAC participated in the design of the study, performed and analyzed immunological and mycobacteriological tests, carried out antigen testing, collected epidemiological data and wrote the manuscript. RKA carried out antigens testing and critical comments of manuscript. CAR carried out mycobacteriological diagnostic, performed immunological test and collected epidemiological data. KNB provided TB antigens Rv0754, Rv0978c, Rv1917c. RR contributed to the design of the study. RS conducted statistical analysis on cytokine production. NKV contributed with statistical analysis on cytokine production. SEH provided critical comments to the manuscript. AZ provided critical comments on the manuscript and provided value information concerning data interpretation. LPG participated in the design of the study, carried out mycobacterial diagnostic, data analysis and manuscript revision. MM participated in the design of the study, data analysis, and helped to draft the manuscript. All authors read and approved the final version of the manuscript.

## Pre-publication history

The pre-publication history for this paper can be accessed here:

http://www.biomedcentral.com/1471-2334/13/125/prepub

## Supplementary Material

Additional file 1 Supplementary materials. Click here for file
