# Supplementary material for: Differential cellular recognition pattern to M. tuberculosis targets defined by IFN-γ and IL-17 production in blood from TB + patients from Honduras as compared to health care workers: TB and immune responses in patients from Honduras
Source: BMC Infect Dis. 2013 Mar 6;13:125. doi: 10.1186/1471-2334-13-125 (PMC3599548; doi:10.1186/1471-2334-13-125)

Supplementary online Material

Supplementary Tables S1a-c, S2-4

Supplementary Figures S1-S2

Supplementary Tables

Supplementary Tables S1a-c Descriptive statistics concerning IFN-γ and IL-17 production in groups 1-3.

S1a. Descriptive statistics of antigens showing significant difference in IFN-γ levels between groups 1 (TB patients), 2 (patients with other pulmonary diseases) and 3 (negative TB cases but highly exposed).

	**Group 1**			**Group 2**			**Group 3**		
Antigen	Median	Mean	S.D	Median	Mean	S.D	Median	Mean	S.D
**SEA/B**	78	169.74	192.12	450	294.9	193.89	450	345.28	173.33
**Rv1886c pep**	0	34.24	94.53	32	89.15	152.6	0	136.24	175.65
**Rv2958c**	0	1.39	4.9	0	4.6	19.83	0	31.14	95.73
**Rv2962c**	0	2.71	12.35	0	6.14	22.14	0	32.14	91.37
**Rv3347c**	0	3.18	12.94	0	9.46	43.95	0	28.9	53.4
**Rv3804c**	23	81.52	137.96	64	171.78	194.95	232	235.82	221.97
**Rv1886c**	0	46.96	102.36	66	156.37	183.79	271	235.47	201.92

S1b – Descriptive statistics of antigens showing significant difference in IL-17 levels between groups

	**Group 1**			**Group 2**			**Group 3**		
**Antigen**	Median	Mean	S.D	Median	Mean	S.D	Median	Mean	S.D.
**Rv1886c**	0	8.04	16.81	9	28.38	41.42	24	39.71	46.64
**Rv0288**	0	0	0	0	2.41	4.77	23	34.67	41.74
**Rv0978c**	0	0	0	0	2.41	4.77	23	34.67	41.74
**Rv1917c**	0	0.6	1.34	0	5.41	8.73	22	21.67	10.5

S1c Antigens showing significant difference in IFN-gamma levels between groups based on ANOVA

**Antigen**	**P val**
SEA/B	0.0001
Rv1886c	0.0175
Rv2958c	0.0126
Rv2962c	0.0123
Rv3347c	0.0316
Ag85A	0.0284
Ag85B	0.0020

Antigens showing significant difference in IL-17 levels between groups based on ANOVA

**Antigen**	**P val**
Ag85B	0.0216
TB10.4	0.0242
Rv0978	0.0022
Rv1917	0.0053

Table S2

Group	Status	Description
1	TB+HIV-	TB cases: Culture and Acid Fast staining positive
2	TB-HIV-	Symptomatic respiratory: Culture, AF-S and QTF negative
3	TB-HIV-	Maybe latent TB: Culture & AFS negative, QTF pos
4	TB-HIV-	Symptomatic respiratory: Previous history of TB, currently negative for culture and AFS
5	TB-HIV-	No symptoms, Health workers Exposed to mycobacteria, TB negative

Table S3 Statistically different IFN-gamma responses in groups 1 – 5

Antigen	Groups	P-value
SEA/B	Group1 vs Group2	0.001
SEA/B	Group1 vs Group3	0.017
SEA/B	Group1 vs Group5	< 0.001
Rv2962c	Group1 vs Group5	0.042
Rv2962c	Group2 vs Group5	0.046
Rv2962c	Group3 vs Group5	0.047
Rv3804c	Group1 vs Group5	0.009
Rv3804c	Group2 vs Group5	0.043
Rv3804c	Group4 vs Group5	0.011
Rv3875	Group2 vs Group3	0.002
Rv3874	Group1 vs Group3	0.025
Rv3874	Group2 vs Group3	< 0.001
Rv3874	Group2 vs Group5	0.011

Table S4 Statistically different IL-17 responses in groups 1 – 5

Antigen	Groups	P-value
Rv3804c	Group1 vs Group5	0.019
Rv3875	Group1 vs Group5	0.027
Rv3874	Group1 vs Group3	0.008
Rv3874	Group2 vs Group3	0.005

Supplementary Figure S1. Differential IFN gamma production in groups 1-5

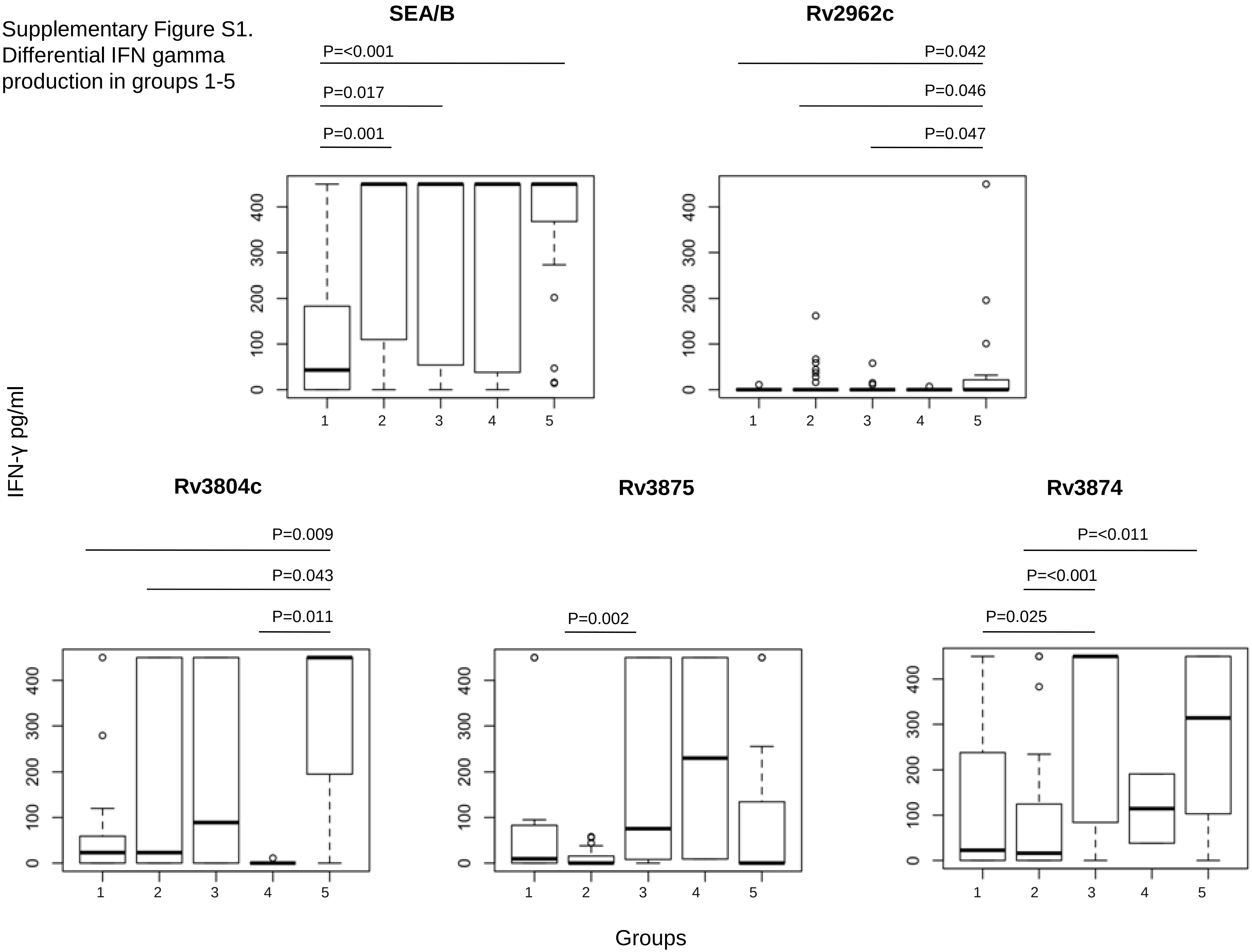

Supplementary Figure S2.
Differential IL-17 production
in groups 1-5

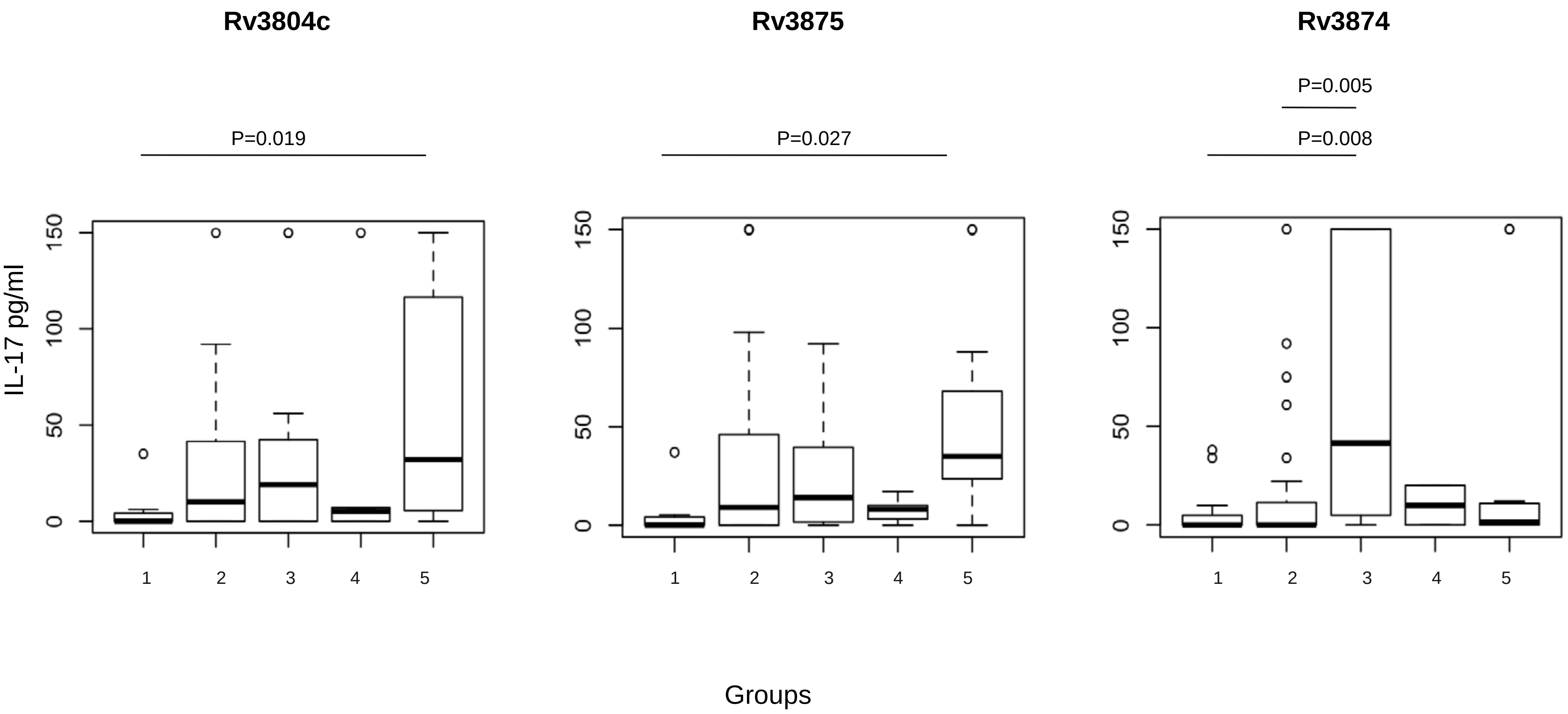

Supplement: Additional file 1 — Supplementary materials. [file 1471-2334-13-125-S1.pdf]
